# Decreased Interactions between Calmodulin and a Mutant Huntingtin Model Might Reduce the Cytotoxic Level of Intracellular Ca^2+^: A Molecular Dynamics Study

**DOI:** 10.3390/ijms22169025

**Published:** 2021-08-21

**Authors:** Sanda Nastasia Moldovean, Vasile Chiş

**Affiliations:** 1 Faculty of Physics, Babeş-Bolyai University, Str. M. Kogălniceanu 1, RO-400084 Cluj-Napoca, Romania; nastasia.moldovean@ubbcluj.ro; 2 Institute for Research, Development and Innovation in Applied Natural Sciences, Babeș-Bolyai University, Str. Fântânele 30, RO-400327 Cluj-Napoca, Romania

**Keywords:** calmodulin, calcium-binding protein, polyglutamine tract, polyglutamine disorders, Huntington’s disease, molecular dynamics

## Abstract

Mutant huntingtin (m-HTT) proteins and calmodulin (CaM) co-localize in the cerebral cortex with significant effects on the intracellular calcium levels by altering the specific calcium-mediated signals. Furthermore, the mutant huntingtin proteins show great affinity for CaM that can lead to a further stabilization of the mutant huntingtin aggregates. In this context, the present study focuses on describing the interactions between CaM and two huntingtin mutants from a biophysical point of view, by using classical Molecular Dynamics techniques. The huntingtin models consist of a wild-type structure, one mutant with 45 glutamine residues and the second mutant with nine additional key-point mutations from glutamine residues into proline residues (9P(EM) model). Our docking scores and binding free energy calculations show higher binding affinities of all HTT models for the C-lobe end of the CaM protein. In terms of dynamic evolution, the 9P(EM) model triggered great structural changes into the CaM protein’s structure and shows the highest fluctuation rates due to its structural transitions at the helical level from α-helices to turns and random coils. Moreover, our proposed 9P(EM) model suggests much lower interaction energies when compared to the 45Qs-HTT mutant model, this finding being in good agreement with the 9P(EM)’s antagonistic effect hypothesis on highly toxic protein–protein interactions.

## 1. Introduction

The polyglutamine (polyQ) tract length of the mutant Huntingtin protein (m-HTT) is generally associated with the disease’s onset and its severity. The physiological Huntingtin protein is expressed in almost all body tissues and has a molecular weight of 348 kDa. The polyQ tract is located at the protein’s N-terminal domain, which is followed by the proline-rich domain (PRD) with a protective anti-aggregation role and a direct involvement in protein–protein interaction complexes [1]. Although the connection between Huntingtin’s (HTT) structure and its function is not yet fully understood [2,3], the accumulation of insoluble aggregated N-terminal fragments of m-HTT together with their associated proteins, leads to loss of interruptions in the extended HTT sequence [4] and a wide range of cell pathologies related to gene modifiers [5,6,7].

The genetic defect in Huntington’s disease (HD) is characterized by the expansion of the CAG trinucleotide repeats at the protein’s N-terminal domain. For normal controls, which are considered as wild-type structures (wt-HTT), the polyQ tract (CAG trinucleotide translates the glutamine amino acid) consists of less than 35 CAG repeats, while the m-HTT is associated with a higher number of trinucleotide repeats [8].

The complex subcellular localization of the HTT structures triggers environmentally dependent conformational changes. Therefore, increasing evidence suggests that phosphorylation at HTT-exon1’s level via specific kinases (Akt, ERK1, and Cdk5) [9] plays a crucial role in structures’ regulation, toxicity levels, and can affect cellular properties not only at the N-terminal domain but also on the full-length HTT sequence [10]. In HD brains or cell culture models of HD, the N-terminal domains also undergo other post-translational modifications such as acetylation. Moreover, the N-terminal regions may also be modified via ubiquitination and sumoylation [10]. For example, the insertion of mutations that mimic a phosphorylated protein, at both Ser-13 and Ser-16, has been shown to modify the levels of ubiquitination and acetylation, to influence HTT-exon1’s clearance and to increase the HTT nuclear localization [11,12].

Biochemical analysis of an HD rat brain also confirmed direct interactions between HTT structures and other interacting partners involved in cellular metabolism, intracellular signaling, and endocytic processes partly associated with clathrin-coated vesicles [13]. Since wild-type HTT structures are crucial in embryogenesis, through calcium regulations [14] and ATP transfer within cells [15], damages in HTT-related pathways may be caused not only by mutant HTT structures’ accumulation but also by the loss of function of the wild-type HTT proteins [15].

Several neurodegenerative disorders with amyloid-like fibrils implications, such as Alzheimer, Parkinson, or Huntington diseases, are associated with great alterations in calcium homeostasis [16]. Additionally, these alterations of Ca^2+^-dependent signaling pathways are mainly present at the early stages of the previously mentioned diseases [16,17].

Ca^2+^ binding proteins are known to play essential roles in enzyme activation [18], gene expression, and proliferation [19]. Although their binding kinetic mechanisms remain unclear [20], they present a great impact on cell death processes by disturbing the Ca^2+^ homeostasis mechanisms [19,21,22]. Higher concentration levels of Ca^2+^ into the neuronal cytosolic environment were found in mutant HD mice models [23,24], where Ca^2+^-binding proteins’ expression and functioning are being altered [24].

The neuroprotective effects are particularly present in large-weight-protein complexes [25,26], where the activation of a Ca^2+^-activated cysteine protease is an almost irreversible process due to the increased cytosolic Ca^2+^ concentrations. Menzies et al. have demonstrated in *Drosophila* HD models that the inhibition of this cysteine protease called calpain has a positive effect on m-HTT’s aggregation and toxicity prevention [27]. This prevention is being outlined by an overexpression of calpain inhibitors that leads to an increased number of autophagosomes, facilitating the recycling mechanisms of dysfunctional or unnecessary cellular components [24,27]. At the genomics level, several studies [24,28,29,30,31] have revealed differences in the mRNAs of the genes, for discrete HD mouse models and patients, which are responsible for intracellular Ca^2+^-regulation proteins’ codification, as well as calcium-binding proteins such as calmodulin (CaM), calbindin, hypocalcin, and several particular receptors such as ryanodine receptor type 1 and voltage-gated Ca^2+^ channels [28,29,30,31].

It has been shown that m-HTT and calmodulin, a calcium-mediated protein that senses the calcium levels and activates particular signals to calcium-dependent or -sensitive enzymes, co-localizes with HTT’s inclusions in the cerebral cortex [25,32] with a direct effect on the dorsal striatum and other basal ganglia [33]. Moreover, the HTT mutants with an extended polyQ tract present higher affinity for CaM when compared to the wild-type HTT models, leading to the formation of monomeric HTT structures and further stabilization of the pre-formed m-HTT aggregates [19]. An excessive and uncontrolled influx of Ca^2+^ through the NMDA receptors has a direct effect on mitochondria, which plays the most important role in Ca^2+^ homeostasis in HD, where it disrupts the membrane potential, releasing cytochrome C and promoting cell death [34,35]. In addition, impaired calcium metabolism was observed in cardiomyopathy related to HD, where the cellular arrhythmia was accompanied by increased activity of Ca^2+^ and accordingly to calmodulin-dependent protein kinases [36]. Moreover, the intracellular calcium bindings by calmodulin could be crucial for proper cellular metabolism not only in CNS but also in all HD-affected tissues [37].

**Figure 1:**
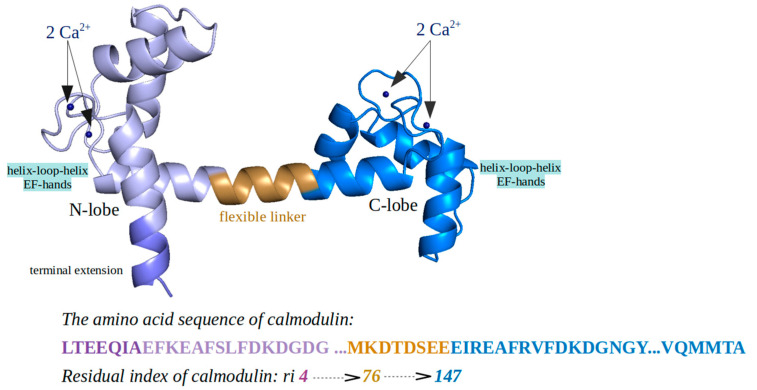
Three-dimensional (3D) structure of calmodulin protein.

Calmodulin is a highly conserved member of the EF-hand proteins family (Figure 1). Its selective binding mechanism to calcium ions corresponds to the helix-loop-helix structural motifs [38,39,40]. Calmodulin forms two structurally homologous globular (N- and C-terminal lobes) domains related to the helical binding motifs, which are connected by a central *α*-helical (of eight turns), flexible, and solvent exposed linker [41,42]. Each of the two domains bind two calcium ions (each EF-hand can bind only one calcium ion), resulting in a total of four Ca^2+^ ions responsible for additional conformational changes such as helical rearrangements on both CaM terminal domains [43]. Upon Ca^2+^ binding, a short region in the middle of the helical linker collapses, which leads to a reorientation of the terminal lobes (from antiparallel to orthogonal orientation) and enables side-chain–side-chain target peptide interactions [44]. In contrast, it has been demonstrated that the apo-CaM model (unbound to the Ca^2+^ ions) is able to bind different other protein targets [45,46].

The structural transition from closed-to-open CaM conformation caused by the helical reorientation within the four EF-hands involves significant repacking of their hydrophobic cores [47]. In its open configuration, the EF-hand domain becomes susceptible to different binding targets by exposing large hydrophobic side-chain clusters [48,49,50].

Although intrinsically disordered proteins are known to be structurally unstable in unbounded states but frequently undertake folded ensembles upon their interactions with specific interacting partners [51], the present study focuses on the direct interaction between CaM structure bound to four Ca^2+^ molecules and three different HTT models (a wild-type structure considered as control model and two mutants). This approach relies on our own ensemble of mutant model (the 9P(EM)) that, by all means, cannot cover the range of its potential and accessible structural states. Considering this, our first attempt is to explore the dynamic involvement of the mutant HTT model upon its interactions with calmodulin with a further analysis of the CaM’s potential structural changes. Moreover, our assumption is that the differences between the HTT models should involve specific structural changes in the CaM’s structure and that the dissimilarity between the wt-HTT structure and the mutant HTT in their interactions with CaM protein will provide further information related to the proposed kinetic mechanisms of the 9P(EM) mutant HTT model. In addition, considering that alpha-helical components are the most common structural components to many calmodulin-binding peptides, this structural feature should also be presented in our molecular docking poses. However, we are interested in how to avoid these types of helical-like structure interactions; therefore, the 9P(EM) model must have a lower binding affinity in comparison to other calmodulin-binding peptides, as long as those peptides present a toxic overall behavior.

## 2. Results and Discussion

### 2.1. Molecular Docking and Binding Free Energy Calculations

**Figure 2:**
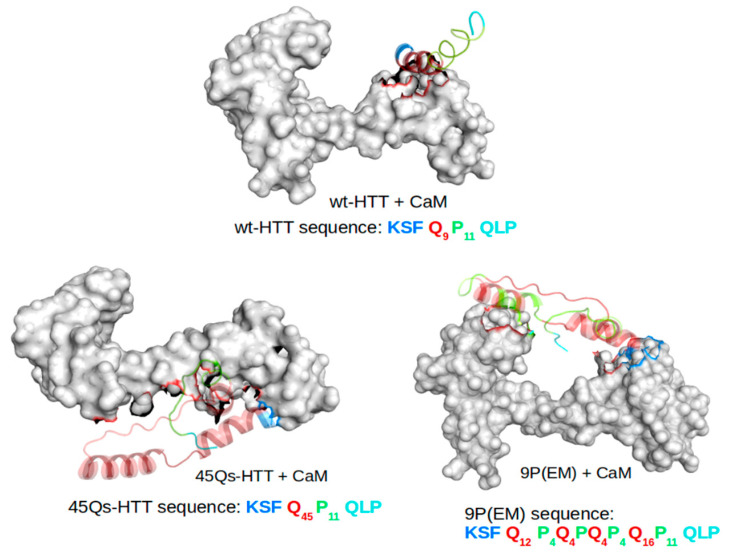
The resulted docked positions for CaM-HTT complexes.

The prediction of binding sites (Figure 2) and the key residues involved in the interactions between HTT models and CaM structure were identified using MM/GBSA calculations from where the generated top 10 docked complexes (exported from multiple docking productions) were analyzed considering their docking scores and binding free energies.

The results show greater structural affinities (higher absolute docking score values) of HTT models to the C-terminal domain of the CaM structure when compared to its N-terminal domain. For the top 10 ranked clusters, the docking scores ranged between −1963.44 and −3722.84. The lower absolute values outlined a binding affinity of the HTTs to the N-lobe of the CaM structure, whereas the highest docking score absolute values corresponded to the binding positions of HTT models to the CaM’s C-terminal lobe (Figure 2).

**Figure 3:**
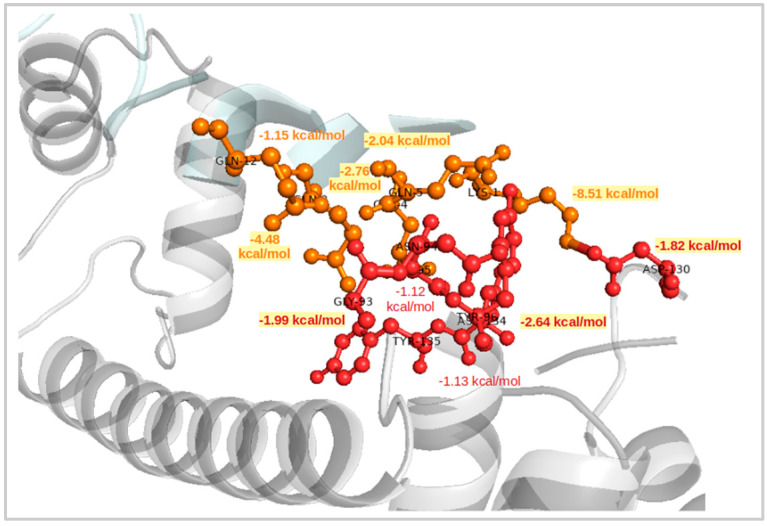
Per-residue contributions for CaM and wt-HTT docked complex and their corresponding binding free energies.

The van der Waals contributions (∆E_vdW_) for the three complexes are nearly seven times lower than the nonpolar solvation free energies (∆G_nonpolar_). For the wt-HTT model (Figure 3), the CaM docked complex presented a total binding free energy of −32.90 kcal/mol from which the polar components (∆E_ele_ and ∆G_polar_) make a positive contribution of 0.77 kcal/mol. The van der Waals interaction energy for this complex was −28.93 kcal/mol and was noted as the lowest absolute value among all complexes. In contrast, the electrostatic interactions for this complex presented the highest absolute value of 985.67 kcal/mol due to large contributions of LYS, ASP, and GLU polar amino acids. The nonpolar component for the same docked complex was −4.73 kcal/mol, being the lowest absolute value obtained for the hydrophobic contributions.

**Figure 4:**
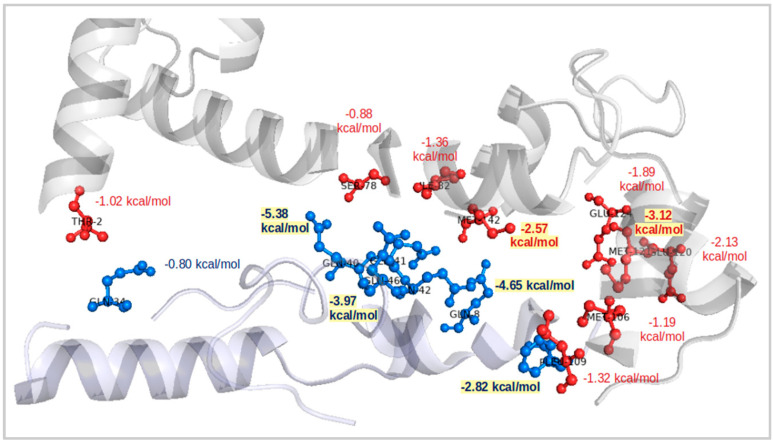
Per-residue contributions for CaM and 45Qs-HTT docked complex and their corresponding binding free energies.

The lowest binding pattern with a total binding free energy value of −23.38 kcal/mol was obtained for the CaM–45Qs HTT (Figure 4) docked complex with similar electrostatic energy value of −962.41 kcal/mol as for the CaM–wt-HTT complex. The polar contributions for this complex presented the highest positive value of 48.94 kcal/mol (with ∆G_polar_ = 1011.35 kcal/mol). For the negative contributions to the binding, the docking results for this complex showed a great importance of the van der Waals interactions with an energy value of −62.73 kcal/mol. In terms of residual components, the LYS-1 residue from the 45Qs-HTT mutant presented an electrostatic energy contribution of −479.08 kcal/mol, which is a related value to the CaM–wt-HTT docked complex (with ∆E_ele_ = −479.44 kcal/mol). From the CaM’s side, most of the GLU amino acids together with ASP and ALA-144 presented the largest contributions for the resulted electrostatic interactions.

**Figure 5:**
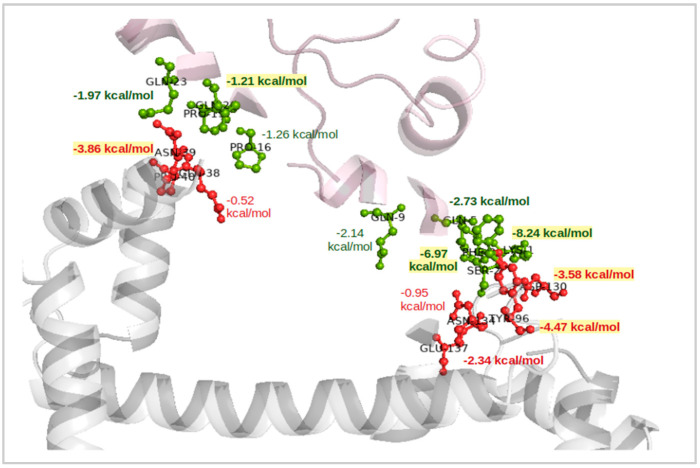
Per-residue contributions for CaM–9P(EM) docked complex and their corresponding binding free energies.

For the CaM–9P(EM) complex (Figure 5), the total binding free energy was −38.31 kcal/mol. The overall contribution values for each energy component were in between the two other docked complexes (CaM with wt-HTT and 45Qs-HTT models). For example, the obtained nonpolar solvation free energy (∆G_nonpolar_) was −6.81 kcal/mol, while for the polar contributions, the ∆G_polar_ and the electrostatic interaction values were −835.31 kcal/mol and −825.03 kcal/mol, respectively. In the same manner as for the 45Qs-HTT model in interaction with CaM, the van der Waals energy value for 9P(EM)–CaM was −41.78 kcal/mol, indicating that indeed, the molecular shape of these interacting partners presents a great influence on the molecular recognition processes of the CaM protein. The electrostatic interaction energy value of the LYS-1 residue from the 9P(EM) model was −459.11 kcal/mol, which is a slightly lower absolute value (by approximately 20 kcal/mol) when compared to the other HTT docked models, while from the CaM’s side, the residual contribution remained the same, with ASP and GLU amino acids having the highest contributions on the ∆E_ele_ component.

Considering the docking scores and the most preferred binding orientations, all HTT models showed higher binding affinities for the same part of the CaM structure and also almost the same conformational poses with the HTT’s N-terminal domain (Figure 3) bound to the C-lobe end of the CaM protein. The docking score for CaM and the wt-HTT interacting partner was −2835.94, while for the mutant HTT models, as expected, the docking scores show similar values of −3722.84 for 45Qs-HTT and −3249.42 for 9P(EM). An interesting molecular conformation was observed for the two HTT mutants that seem to adopt “bridge”-like orientations between the two CaM lobes but on opposite sides of the protein (45Qs-HTT binds to the lower part of the protein, while the 9P(EM) model binds to the superior part of CaM).

### 2.2. CaM and wt/m-HTT Interacting Complexes

**Figure 6:**
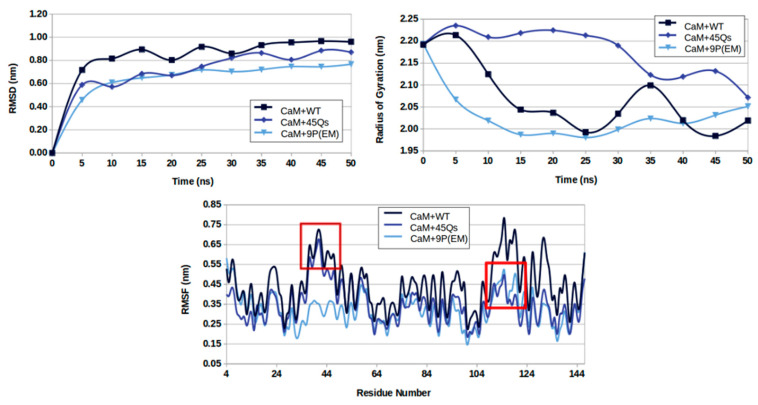
RMSD (**top left**), radius of gyration (**top right**), and RMSF (**bottom**) profiles for CaM protein in its interaction with the HTT models.

The analysis of the interacting complexes show that all systems reached their stability after 10 ns of MD run (Figure 6). The highest RMSD profile for the CaM structure with an average value of 0.80 nm was observed for its interactions with the wt-HTT model. In addition, for the same complex, in the last 10 ns of simulation, the highest obtained average value was 0.966 nm. In contrast, for the systems with 45 Qs and 9P(EM) interacting partners, CaM showed similar average values of 0.68 nm and 0.62 nm, respectively. The lowest RMSD profiles were observed for the CaM–9P(EM) complex where the average maximum values ranged between 0.71 and 0.76 nm.

The higher variations of RMSD values for the CaM in interaction with the wt-HTT model are most certainly caused by the higher flexibility of the wt-HTT structure, consequently promoting an increase in the number of degrees of freedom of the CaM protein. These findings are consistent with the gyration behavior of the complex.

Starting from a gyration value of 2.19 nm, the CaM protein’s Rg profile decreased to 2.07 nm when interacting with the wt-HTT model. Although the minimum obtained Rg average value of 2.03 nm was observed for CaM-9P(EM) complex, for the other mutant (45Qs-HTT model), the CaM structure shows the highest Rg average value of 2.18 nm.

As expected, considering that the CaM dynamics show high RMSD variations in its interaction with the wt-HTT model, the RMSF values for the same interacting complex give a maximum RMSF average value of 0.44 nm. The lowest RMS fluctuation values were obtained for CaM in interaction with the 9P(EM) model where the averaged values over multiple runs converged to 0.32 nm. A comparable behavior was observed for the CaM protein with the 45Qs-HTT interacting partner with an average RMSF value of 0.35 nm.

The number of CaM’s residues involved in the highest fluctuation rates was around 15 amino acids from the protein’s N-lobe and 25 amino acids from the CaM’s C-lobe. In the complex formed with the wt-HTT model, the CaM’s residues with highest RMSF values were ASN-42, PRO-43, THR-44, GLU-45, and ALA-46 from the N-lobe side, while from the C-lobe end, the mostly involved residues were ASN-111, GLY-113, LYS-115, LEU-116, THR-117, GLU-119, ILE-130, ASP-131, and ALA-147. For the CaM protein’s interactions with the 45Qs-HTT mutant, the involved residues from the CaM’s N-terminal lobe were almost the same as in the interactions with the wt-HTT model. On the other hand, for the other two interacting partners (45Qs and 9P(EM) HTT mutants), the CaM’s residues fluctuations were lower at the C-lobe end of the structure. Overall, the same involved residues in all three complexes showed the lowest fluctuation rates for both CaM’s lobes in its interaction with the 9P(EM) mutant model.

**Figure 7:**
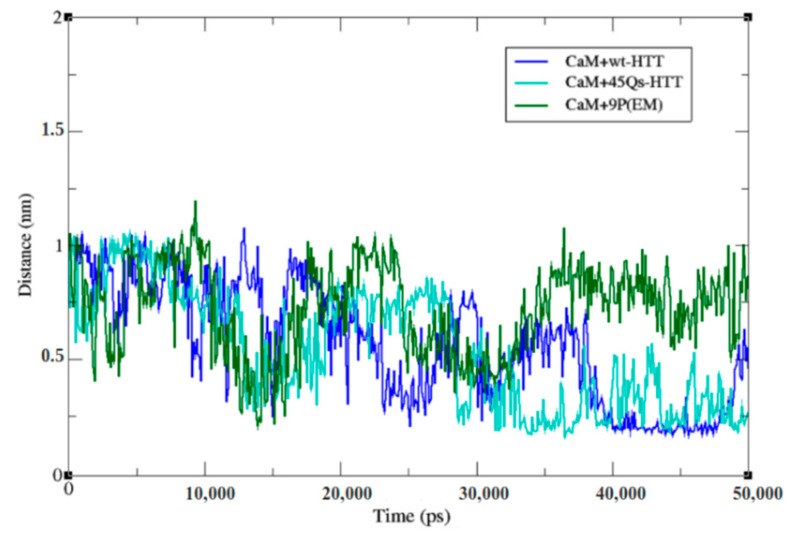
Minimum distance between the two lobes of the CaM structure.

The structural changes with respect to the CaM lobes were studied using a minimum distances analysis tool. The results (Figure 7) showed that the CaM protein in its interactions with the wt-HTT model had the shortest average distance between the lobes of 0.54 nm. This value is correlated to the CaM’s gyration behavior with the same interacting partner with an average value of 2.07 nm. On the other hand, the gyration values for this complex were not the lowest ones. Interestingly, the lowest gyration profile, which means the highest compactness level, was observed for the CaM protein in its interactions with the 9P(EM)-HTT model, where the minimum distance value between the CaM’s lobes represented actually the maximum average value of 0.77 nm. An intermediate minimum distance average value between the N-lobe and C-lobe of 0.57 nm was obtained for the CaM-45Qs HTT interacting complex. These findings might involve the fact that the CaM structure shows similar structural behavior and that the helical linker is less flexible in its interactions with the wt-HTT and 45Qs-HTT models when compared to the interactions with the 9P(EM) model.

The visual inspection on the MKDTDSEE part of CaM’s sequence (the helical linker structural component) suggests that the largest average distance between the CaM protein’s lobes corresponds to drastic structural changes at that level from helical configurations to loop configuration. An explanation for the lower CaM gyration profile is that after these structural transitions occur, the N-lobe and C-lobe of CaM get much closer to each other.

### 2.3. Structural Changes of wt-HTT, 45Qs-HTT, and 9P(EM) Models

A higher radius was observed in the gyration profiles for both HTT mutant models with the same average Rg value of 1.25 nm. The starting compactness value for the mutants was around 1.45 nm, which is a value that decreased with almost 0.3 nm in the first 5 ns of the simulation time. For the wt-HTT, also because of the short polyQ sequence and consequently lower number of helical components, the Rg average value was 1.22 nm and remained constant during the simulation, presenting the smallest variations among all HTT models.

**Figure 8:**
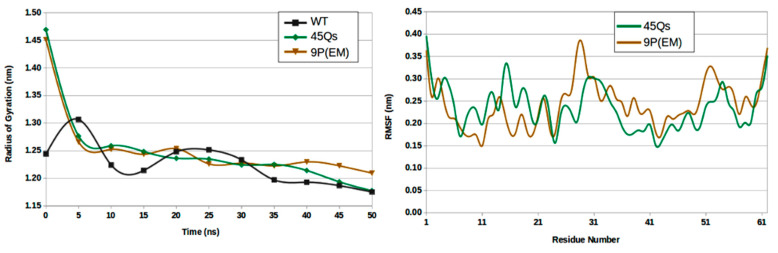
The radius of gyration (**left**) and RMSF (**right**) profiles for HTT models in their interaction with CaM.

A maximum RMSF average value of 0.34 nm was obtained for the wt-HTT, while the mutants presented similar fluctuation behavior (Figure 8). The minimum RMSF values ranging between 0.15 and 0.40 nm were related to the HTT mutants with a few particularities: for the 45Qs-HTT mutant model, the glutamine residues closer to the N-terminal domain presented higher fluctuation rates due to the larger content of random coils and bends, while for the 9P(EM), the structural transformations became more abundant for the GLN residues starting from the middle of the helix.

**Figure 9:**
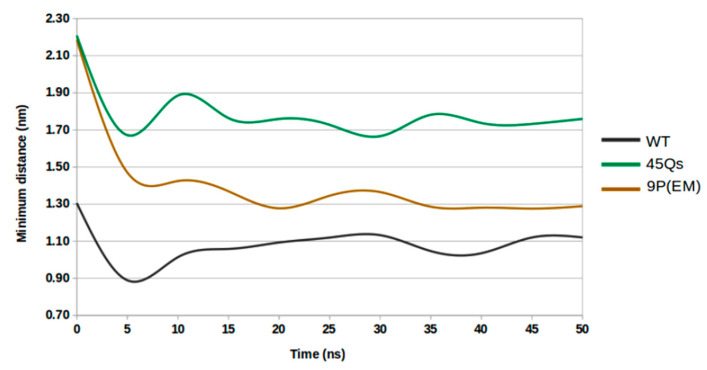
Plot of the minimum distances between the edges of the helix for the HTT models as a function of time.

The minimum distance measurements (Figure 9) between the helical edges suggest a decrease by 0.18 nm at the end of the simulation for the wt-HTT model, indicating an overall preservation of its helical content. The 45Qs-HTT structure presents a minimum distance average value of 1.79 nm between the helical edges, while for the 9P(EM) mutant model, the minimum average distance was 1.42 nm. Both mutants had a starting helical distance of approximately 2.20 nm and show a significant decrease to 1.66 nm after 30 ns for the 45Qs-HTT model, whereas for the 9P(EM), after just 20 ns, the distance between the edges of the helix was 1.27 nm. These findings are well correlated to the helical disruption rates that are higher for the proposed 9P(EM) mutant model [52] when compared to the 45Qs-HTT model.

**Figure 10:**
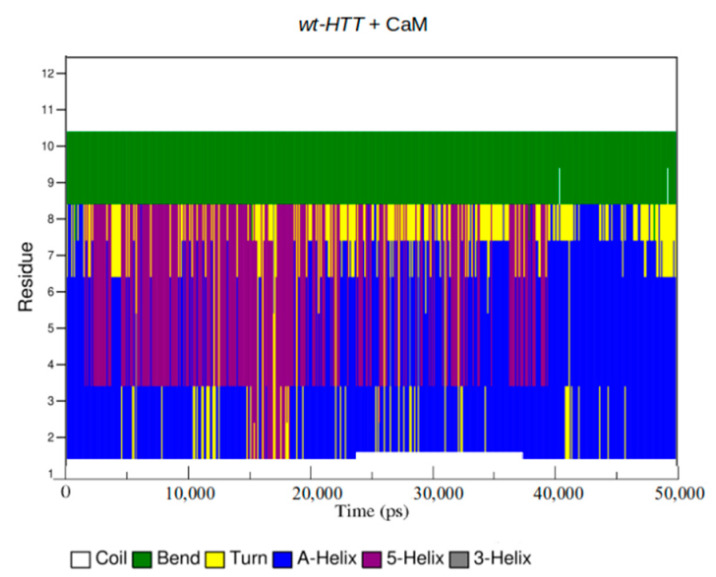
DSSP plots for secondary structure transitions between residue numbers 4 and 12 for the wt-HTT model.

**Figure 11:**
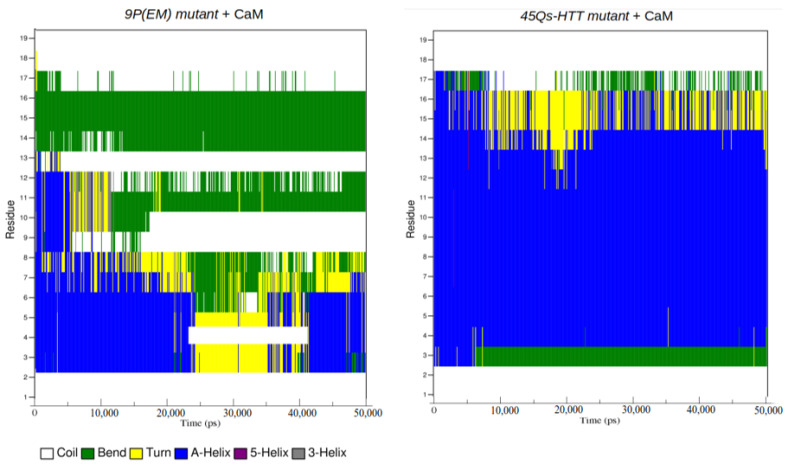
DSSP plots for secondary structure transitions between residue numbers 15 and 33 for the two mutant HTT models.

In order to obtain an accurate comparison between the structural components of each HTT model, we have identified the groups of atoms that form the insoluble α-helices and hardly soluble five-helical components with the purpose of monitoring their structural transformations during the 50 ns simulation time. The wt-HTT model shows during the first 20 ns and the last 10 ns of simulation an increased number of helical configurations, the number of residues alternating between α-helical and five-helical components (Figure 10 and Figure 11 and Figure S1 in Supplementary Materials). For the 9P(EM) mutant HTT model, the number of helical configurations significantly decreases when compared to the mutant model with 45Qs. A clear structural transition from helices to random coils can also be seen for the 9P(EM) model in Figure 11. For both HTT mutants, after 20 ns of simulation, the extended content of helices is being replaced by turns, which are a secondary structure element considered to be essential for the folding processes by allowing specific interactions between other secondary structure components [53]. Moreover, turns can also be found as fully unstructured segments in multi-domain proteins or in random coil components corresponding to partially structured complexes [54].

### 2.4. Interaction Patterns for CaM-HTT Complexes

**Figure 12:**
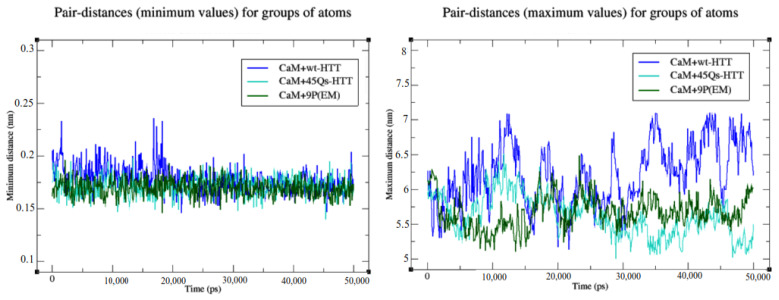
Pairwise distances between CaM and the HTT models.

The maximum pairwise distances between CaM residues and HTT models describe the long-range contacts between the CaM’s N-lobe and the HTT residues (Figure 12, right). Between 10 and 33 ns, the residue contacts of CaM and the wt-HTT model slightly decreased from 6.31 to 5.62 nm, implying minor structural deformations of the helical linker. From 35 ns until the end of the simulation, the wt-HTT reoriented itself in order to form closer contacts between the N-terminal residues (KSF sequence) and the CaM’s C-lobe end (KDGNGYI and VDEMIRE sequences). The same structural orientation (with the N-terminal domain of the HTT structure toward the CaM’s C-lobe end) was observed for the 45Qs-HTT mutant model with the observation that GLN26-33 residues, part of the mutant helical content, got closer to the N-lobe of the CaM protein after 35 ns of simulation due to the HTT mutant’s extended polyQ tract. For this model, the average maximum distance was 5.63 nm. A similar behavior was observed for the CaM-9P(EM) complex that presents the lowest maximum distance value of 5.77 nm between the structures, mainly because of the high flexibility rates of the CaM’s helical linker.

It is important to note that regardless of the mutants’ structural changes, they never left the C-lobe docked pocket of the CaM against their mutant HTT N-terminal domain. The minimum distances between CaM and HTT models correspond to their interactions with the C-lobe end of the CaM with an average distance of 0.17 nm for the wt-HTT and for the 9P(EM) model and with a slightly increased value of 0.18 nm for the 45Qs-HTT model.

The Lennard–Jones and Coulombic interaction energies were extracted using the *gmx energy* tool (Table S1, Supplementary Materials). The short-range interactions were carried out using energy groups (CaM and each HTT model) and by invoking the *-rerun* option to recalculate these energies from the corrected trajectories. Considering that the energetic components cannot be experimentally quantified, the total interaction energies were calculated (in kcal/mol) and considered for further analysis.

Our previous study [52] has demonstrated that the 9P(EM) model with specific key-point mutations at the edges and in the middle of the helix presents an antagonistic effect due to its drastic structural changes from helical configurations to random coils; therefore, we hypothesized that it might also present lower interaction energies with the CaM protein. Consequently, this study confirms that the total interaction energy between CaM and the wt-HTT model is −130.11 kcal/mol and, as expected, a much higher total interaction energy value of −313.87 kcal/mol was obtained for the CaM’s interactions with the 45Qs-HTT structure. Moreover, an intermediate interaction energy averaged value of −238.95 kcal/mol was observed for the 9P(EM) model.

## 3. Materials and Methods

The MD trajectories described in the present work were carried out by using the GROMACS [55] v2018.1 simulation package and GROMOS43a2 force field [56] suitable for biomolecular systems’ description due to the changes in torsion angles and the third-neighbor van der Waals interaction parametrization. The analysis components described by RMSD, RMSF, radius of gyration, distances, and secondary structure profiles were performed using the analysis tools incorporated in the Gromacs package. The output trajectories were analyzed using PyMol molecular visualization program [57].

### 3.1. Preparation of CaM, HTT, and CaM–HTT Complexes

**Figure 13:**
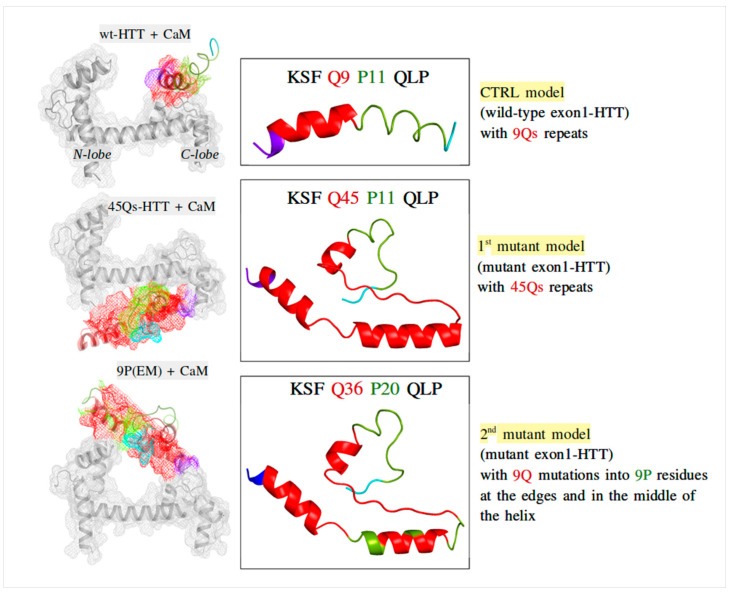
The input HTT models (**right**) with their corresponding docked CaM–HTT complexes (**left**).

For all three systems, the 3D structure of CaM (Figure 13) was obtained from the Protein Data Bank database, from where the structure refined at 1.7 Å resolution (PDB code: 1CLL) [58] with the four calcium ions was used for further investigations. The ethanol and the water molecules surrounding the EF-hands were removed. The final CaM input structure consisted of 144 residues and 1133 atoms.

The HTT input models (Figure 13) were considered as follows: the amino acid sequence used for the control model (wt-HTT) was KSFQ_9_P_11_QLP; for the first mutant, we used the sequence KSFQ_45_P_11_QLP, and for the second mutant structure, we used the 9P(EM) model analyzed in our previous published paper [52]. The 9P(EM) structure is characterized by specific key-point mutations along the 45Qs tract, with 9 glutamine residues mutated into proline (P) residues at the edges (4-4 residues) and in the middle (1 residue) of the helical content [52].

Prior to multiple MD simulations, the CaM–HTT complexes were subjected to a molecular docking technique in order to obtain the predicted binding sites and to identify the key residues involved in CaM–HTT model interactions (Figure 13). The docked complexes were obtained using the HawkDock [59,60,61] server and analyzed using UCSF Chimera [62] interactive visualization and analysis software.

### 3.2. Molecular Dynamics of CaM–HTT Complexes

The complexes were solvated with an SPC water model [63] in a cubic box of 1 nm distances on all axis and with periodic boundary conditions applied. Afterwards, the systems were neutralized with counter ions and energy was minimized for 50,000 steps without restraints using the steepest descent minimization algorithm. Next, the systems were equilibrated using a modified Berendsen thermostat and barostat for 100 ps in an NVT ensemble followed by another 100 ps in an NPT ensemble at 310 K temperature and 1 bar pressure.

Long-range electrostatic interactions were described using the particle-mesh Ewald method [64] with a cut-off of 12 Å. In addition, the LINCS algorithm [65] was used for all h-bond holonomic constraints. Finally, a 2 fs time integration step was considered for multiple 50 ns MD productions.

## 4. Conclusions

The presented results support our hypothesis that the 9P(EM) structure might represent a suitable model for protein–protein interaction inhibition with respect to CaM, and it may slow down the cytotoxic processes by suppressing the intracellular calcium levels. The molecular docking analysis showed higher binding free energy contributions for LYS, GLU, and ASP residues of the CaM structure. Moreover, the highest residual affinity for all the three HTT models was observed for LYS-1 residue from the N-terminal domain. In addition, all the HTT models show the highest binding affinities for the C-lobe end of the CaM structure. Regarding the 45Qs–HTT model, the highest docking score was observed when the 45Qs structure was oriented with the loop configurations towards, which implies the formation of a bridge-like structure that connects the two CaM’s lobes. When compared to the other HTT models (wt-HTT and 9P(EM)), this arrangement presents a slightly lower docking score, while the interactions between calmodulin and the 45Qs–HTT model still exist due to strong van der Waals interactions and the higher electrostatic energy values of the N-terminal domain. In addition, the resulted lower binding pattern can also be correlated to the highest positive polar contributions of the 45Qs–HTT model.

Consequently, at a molecular level, the expanded polyQ tracts can increase their affinities to other mutant protein interactions. Therefore, we can assume that mutant HTTs trigger the formation of more avidly and toxic complexes regardless of the presence or absence of calcium, and considering that the wild-type HTT structures interact with calmodulin only in the presence of calcium.

The highest compactness level of CaM interacting with the 9P(EM) involves drastic structural changes of the CaM’s helical linker that might increase the chance for the CaM’s lobes to overlap. The RMS fluctuation rates are higher for the 9P(EM) model, while the minimum distance values between the edges of the helix, for the same model, significantly decreased, implying drastic structural transitions from α-helices to turns and highly soluble random coils. Considering these changes, we can relate the structural behavior of our proposed model to its interaction energies that were much lower in comparison to the 45Qs–HTT mutant.

The disruption of mutant HTT–CaM interaction mechanisms, by using our proposed 9P(EM) mutant model, might also represent a potential therapeutic target against HD. As a result, the reduction in mutant HTT aggregates’ accumulation will directly impact the uncontrolled intracellular Ca^2+^ uptake levels and can normalize the mitochondrial defects associated with the overexpression of mutant HTT structures. All these protective effects will be considered for further experimental approaches and could serve as a novel biophysical interacting complex for drug development in HD.

## Data Availability

All data generated or analyzed during this study are included in this published article and its Supplementary information files.

## Supplementary Materials

The following are available online at https://www.mdpi.com/article/10.3390/ijms22169025/s1, Figure S1: The secondary structure components in a number of residues for the HTT models during their interactions with CaM protein; Table S1: Lennard–Jones and Coulombic interaction energy components and the total interaction energies between CaM and the HTT models. List of abbreviations.
