# Supplementary material for: Decreased Interactions between Calmodulin and a Mutant Huntingtin Model Might Reduce the Cytotoxic Level of Intracellular Ca2+: A Molecular Dynamics Study
Source: Int J Mol Sci. 2021 Aug 21;22(16):9025. doi: 10.3390/ijms22169025 (PMC8396531; doi:10.3390/ijms22169025)
Supplement: Supplementary file 1 [file ijms-22-09025-s001.zip › Supplementary_Materials.pdf]

International Journal of *Molecular Sciences*

MDPI

Research Article

Decreased Interactions between Calmodulin and a Mutant Huntingtin Model Might Reduce the Cytotoxic Level of Intracellular Ca^{2+}: A Molecular Dynamics Study

Sanda Nastasia Moldovean[1,2] and Vasile Chiş[1,2*]

1 Babeş-Bolyai University, Faculty of Physics, Str. M. Kogălniceanu 1, RO-400084 Cluj-Napoca, Romania
2 Institute for Research, Development and Innovation in Applied Natural Sciences, Babeş-Bolyai University, Str. Fântânele 30, RO-400327, Cluj-Napoca, Romania
* Correspondence: vasile.chis@ubbcluj.ro.

Supplementary Materials

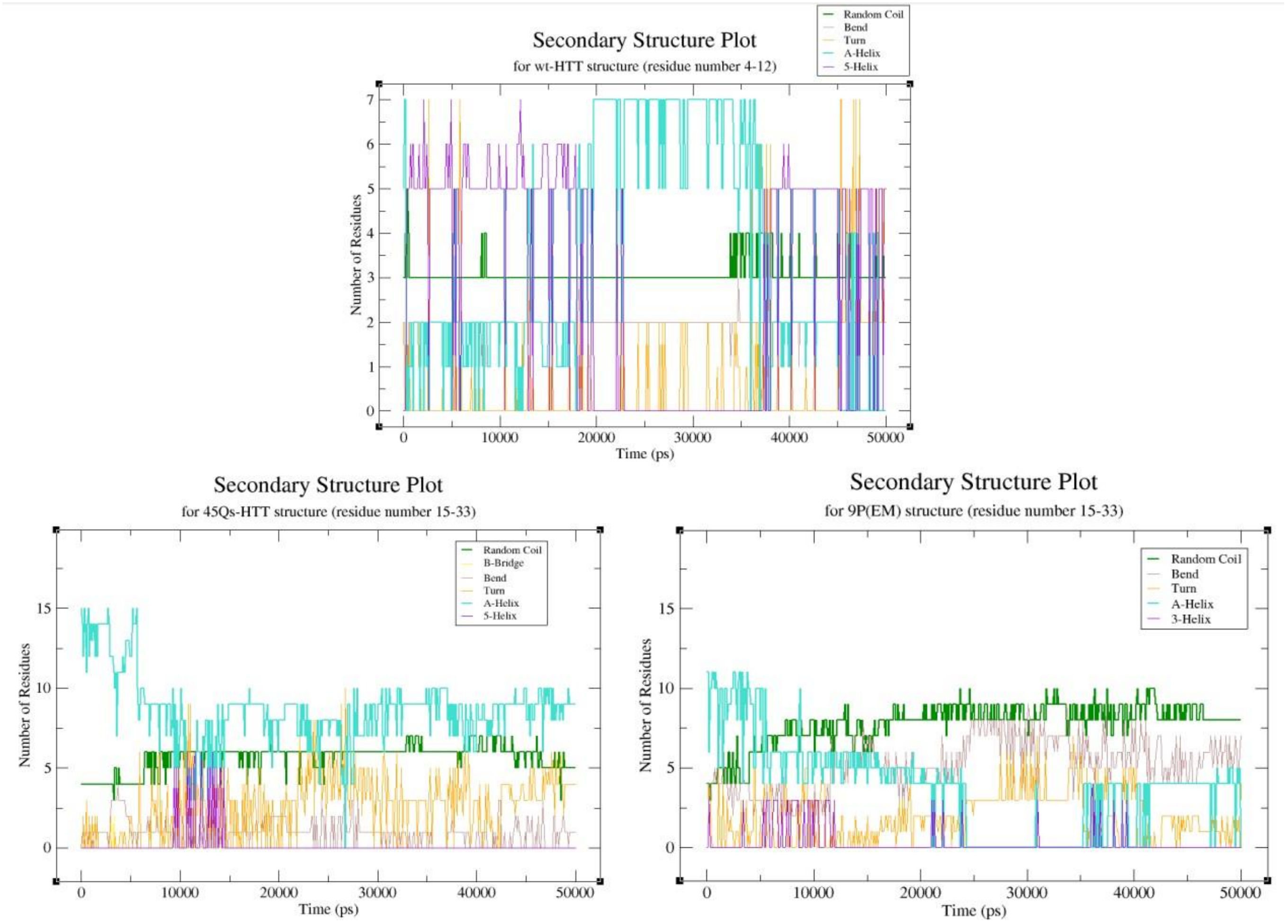

Figure S1. The secondary structure components in number of residues for the HTT models during their interactions with CaM protein.

All of the illustrated plots were obtained for indexed residues using the DSSP tool from the Gromacs v.2018 package.

Table S1. Lennard-Jones and Coulombic interaction energy components and the total interaction energies between CaM and the HTT models.

CaM+wt-HTT		CaM+45Qs-HTT		CaM+9P(EM)	
S-R Lennard-Jones (kcal/mol)	S-R Coulombic (kcal/mol)	S-R Lennard-Jones (kcal/mol)	S-R Coulombic (kcal/mol)	S-R Lennard-Jones (kcal/mol)	S-R Coulombic (kcal/mol)
-64.60	-65.50	-150.64	-163.22	-117.00	-121.95
Total interaction energy: -130.11		Total interaction energy: -313.87		Total interaction energy: -238.95	

List of Abbreviations

polyQ	polyglutamine
Q	glutamine
HTT	Huntingtin
PRD	Proline-Rich Domain
m-HTT	mutant Huntingtin
wt-HTT	wild-type Huntingtin
CAG	Cytosine Adenine Guanine
9P(EM)	9 mutation points at the edges and in the middle of the helix
HD	Huntington's disease
ATP	Adenosine 5'-triphosphate
Ca^{2+}	Calcium ions
mRNA	messenger ribonucleic acid
CaM	Calmodulin
CNS	Central Nervous System
EF-hand	Helix-loop-helix structural motif
MM/GBSA	Molecular Mechanics energies combined with Generalized Born and Surface Area continuum solvation
MD	Molecular Dynamics
RMSD	Root Mean Square Deviation
RMSF	Root Mean Square Fluctuation
Rg	Radius of gyration
DSSP	Define Secondary Structure of Proteins
PDB	Protein Data Bank
SPC	Simple Point Charge
NVT	constant-temperature, constant-volume ensemble
NPT	constant-temperature, constant-pressure ensemble
LINCS	LINear Constraint Solver algorithm
